# A Meta-Analysis for Association of *XRCC1, XRCC2* and* XRCC3 *Polymorphisms with Susceptibility to Thyroid Cancer

**DOI:** 10.31557/APJCP.2021.22.7.2221

**Published:** 2021-07

**Authors:** Mohammad Mandegari, Seyed Alireza Dastgheib, Fatemeh Asadian, Seyed Hossein Shaker, Seyed Mostafa Tabatabaie, Shadi Kargar, Jalal Sadeghizadeh-Yazdi, Hossein Neamatzadeh

**Affiliations:** 1 *Department of Otolaryngology, Head and Neck Surgery, Otorhinolaryngology Research Center, Shahid Sadoughi University of Medical Sciences, Yazd, Iran. *; 2 *Department of Medical Genetics, School of Medicine, Shiraz University of Medical Sciences, Shiraz, Iran. *; 3 *Department of Medical Laboratory Sciences, School of Paramedical Science, Shiraz University of Medical Sciences, Shiraz, Iran. *; 4 *Department of Emergency Medicine, Iran University of Medical Sciences, Iran, Iran. *; 5 *Department of General Surgery, Shahid Sadoughi University of Medical Sciences, Yazd, Iran. *; 6 *Department of Medical Genetics, Shahid Sadoughi University of Medical Sciences, Yazd, Iran. *; 7 *Department of Food Science and Technology, School of Public Health, Shahid Sadoughi University of Medical Sciences, Yazd, Iran.*

**Keywords:** Thyroid cancer, XRCC1, XRCC2, XRCC3, genetic polymorphisms, meta-analysis

## Abstract

**Background:**

We conducted a comprehensive meta-analysis to explore the association of polymorphisms at *XRCC1*, *XRCC2* and *XRCC3* genes with susceptibility to thyroid cancer (TC).

**Methods:**

We searched PubMed, EMBASE, Web of Science, and CNKI for relevant available studies. The pooled odds ratios (ORs) with 95% confidence intervals (CIs) were used to evaluate the strength of the associations.

**Results:**

A total of 67 studies including 17 studies with 6,806 cases and 5,229 controls on *XRCC1* Arg399Gln, 13 studies with 3,234 cases and 4,807 controls on *XRCC1* Arg280His, 13 studies with 2,956 cases and 3,860 controls on *XRCC1* Arg194Trp, five studies with 1,287 cases and 1,422 controls on *XRCC2* Arg188His, 13 studies with 2,488 cases and 3,586 controls on *XRCC3* Thr241Met, and six studies with 1,828 cases and 2,060 controls on *XRCC3* IVS5-14 polymorphism were selected. Polled data revealed that the *XRCC1* Arg399Gln, Arg280His, Arg194Trp, *XRCC2* Arg188His and *XRCC3* Thr241Met and IVS5-14 polymorphisms were not significantly associated with an increased risk of TC. Stratified analyses by ethnicity showed that the *XRCC1* Arg399Gln polymorphism was associated with TC risk in Caucasians, but not in Asians.

**Conclusions:**

Our meta-analysis indicated that the *XRCC1* Arg399Gln, Arg280His, Arg194Trp, *XRCC2* Arg188His, *XRCC3* Thr241Met and IVS5-14 polymorphisms were not associated with risk of TC in the global population. Further well-designed investigations with large sample sizes are required to confirm our results.

## Introduction

Thyroid carcinoma (TC) is the most common endocrine malignancy and accounts for 0.5–1.5% of all cancer cases in the United States (Ortega et al., 2004; Joseph et al., 2018). The incidence of TC has increased globally in recent decades, especially among younger adults. Its incidence and mortality rates are varied by 8–12 fold and 2–6-fold, respectively (La Vecchia et al., 2015; Sierra et al, 2016; Sanabria et al., 2018). In the United States of America (USA), deaths from TC alone account for more deaths than all of the other endocrine malignancies with annual incidence of 6.6% between 2000 and 2009 is the highest among all cancers (Davies et al., 2015; Kim et al., 2020; Yan et al., 2020). TC is more common in women than in men, but men are twice as likely as women to die from this cancer (Rahbari et al., 2010). The Papillary Thyroid Carcinoma (PTC) is the most frequent subtype of thyroid malignancy which constitutes approximately >85% of all cases (Schlumberger and Torlantano, 2000; Baloch and LiVolsi, 2018; Joseph et al., 2018). The etiology and development of TC is a result of complex interactions between genetic and environmental factors (Makazlieva et al., 2016; Boi et al., 2017; Nettore et al., 2018). Continuously exposed to a wide range radiation is a well-established risk factor for TC, which such radiation include certain radiation therapy and radiation fallout from power plant accidents of atomic bombs (Yamashita and Suzuki, 2013; Iglesias et al., 2017; Fiore et al., 2019).

There is increasing evidence suggests that damage to human DNA might initiate the cancer, which caused by external agents such as chemical agents, ionizing radiation and UV (Lange et al., 2011; Barnes et al., 2018). To date, several genetic variations that have a fundamental role in the carcinogenesis of different subtypes of TC have been reported (Xing, 2013; Penna et al., 2016). DNA repair is essential for the maintenance of genomic integrity, which is of primary importance in the general and specialized functions of cells, as well as in the prevention of carcinogenesis (Li et al., 2019). Some genes of the X-ray repair cross-complementing (XRCC) family are an essential part of the BER and homologous recombination (HR) DNA repair pathways responsible for DNA double strand breaks caused by normal metabolic processes and/or exposure to ionizing radiation, and have been reported to be associated with development of TC (Namazi et al., 2015; Cannan and Pederson, 2016; Yan et al., 2016). Previous studies have reported that X polymorphisms of *XRCC1*, *XRCC2*, *XRCC3* DNA repair genes are associated with an increased risk of TC in in different populations (Hu et al., 2013; Jafari Nedooshan et al., 2017). However, no conclusive result has been reported due to the conflicting results among different studies. Therefore, a meta-analysis of all available studies will help to obtain a more convincing result, because some of these studies were based on small sample size, thus, subgroup analysis ethnicity may also yield more meaningful results (Dijkman et al., 2009; Ganeshkumar and Gopalakrishnan, 2013). Here, we performed a meta-analysis of all eligible case-control studies published to date, to assess the association of *XRCC1*, *XRCC2* and *XRCC3* polymorphisms with susceptibility of TC globally.

## Materials and Methods

*Publication Search*

Ethical approval or patient consent was not required because this is a meta-analysis in which all data were extracted from published data. A comprehensive computer search was carried out independently by two authors, in PubMed, Web of Knowledge, Web of Science, Embase, Scientific Information Database (SID), WanFang, VIP, Chinese Biomedical Database (CBD), Scientific Electronic Library Online (SciELO) and China National Knowledge Infrastructure (CNKI) database to collect the case-control studies that investigated the association *XRCC1*, *XRCC2* and *XRCC3* genes polymorphisms with TC risk up to October 01, 2020. Combinations of the following keywords were used in the search: (‘’Thyroid Cancer’’ OR ’’Thyroid Carcinoma‘’ OR ‘’Papillary Thyroid Cancer ‘’ OR ‘’Follicular Thyroid Cancer’’ OR ‘’Hurthle Cell Cancer’’ OR ‘’Medullary Thyroid Cancer’’ OR Anaplastic Thyroid Cancer’’) AND (‘’X-Ray Repair Cross-Complementing Protein I’’ OR ‘’*XRCC1*’’ OR ‘’rs1799782’’ OR ‘’Arg194Trp’’ OR ‘’rs25487’’ OR ‘’Arg399Gln’’ OR ‘’rs25489 OR ‘’Arg280His’’) AND (‘’X-Ray Cross Complementing Group II’’ OR ‘’*XRCC2*’’ OR ‘’Arg188His’’ OR ‘’rs3218536’’) AND (‘’X-Ray Cross Complementing Group III’’ OR ‘’*XRCC3*’’ OR ‘’Thr241Met’’ OR ‘’rs861539’’) AND (‘’Gene’’ OR ‘’Genotype’’ OR ‘’Allele’’ OR ‘’Polymorphism’’ OR ‘’Single Nucleotide Polymorphisms’’ OR ‘’SNPs’’ OR ‘’Variation’’ OR ‘’Mutation’’). In addition, to prevent the loss of any important data, we reviewed the bibliographical references list of retrieved studies, reviews and previous meta-analyses. The whole search process was carried out in English, Chinese, Portuguese, Russian and Persian. When overlapping data on the same cases were included in more than one publication, only the one with the larger sample size was selected.

*Inclusion and Excluding Criteria*

The studies included in the meta-analysis were required to meet the following criteria: 1) Case-control study of TC cases and healthy subjects; 2) studies evaluated the association of polymorphisms at *XRCC1*, *XRCC2* and *XRCC3* genes with TC; 3) provide both genotype and allele distributions inpatients and controls for estimation of combined odds ratios (OR) and 95% confidence intervals (CI); and 4) full text studies on human. Accordingly, Studies were excluded if they: 1) abstracts, reviews, editorials, comments or animal studies; 2) case only studies; 3) linkage studies and family based studies; 4) did not provide the numbers of genotypes; 5) animal and in vitro studies; and 6) contained overlapping data. If the full text article or a study did not published detailed data regarding the genotype distribution in cases and controls, the corresponding authors of the study were contacted for unpublished data.

*Data Extraction*

Two authors worked independently to extract all data from all eligible studies based on the inclusion criteria. Any disagreement was resolved by further discussion until a consensus about valid data was reached. The publication details collected included: first author’s name, year of publication, ethnicity (Asian, Caucasian, African and mixed populations), country of origin, genotyping methods, numbers of cases and controls, frequencies of genotypes in cases and controls, minor allele frequency (MAF) in controls, and Hardy-Weinberg equilibrium (HWE) in controls. The ‘‘mixed’’ group means mixed or unknown populations. Moreover, when publications included sample of more than one ethnicity or population, the data was extracted separately according to ethnicities. The publications did not reported necessary data, as well as genotype frequencies; we contacted the corresponding authors by email to request the missing data.

*Statistical Analysis*

The strength of the association between different polymorphism of *XRCC1*, *XRCC2* and *XRCC3* genes and TC risk was estimated by calculating pooled odds ratios (OR) with 95% confidence intervals (CI). The significance of the summary of pooled data was tested using a Z-test in which P-values less than 0.05 were considered to be statistically significant. The association of the *XRCC1*, *XRCC2* and *XRCC3* polymorphisms with TC risk was evaluated under models, i.e., allele (B vs. A), homozygote (BB vs. AA), heterozygote (BA vs. AA), dominant (BB+BA vs. AA) and recessive model (BB vs. BA+AA), respectively. The between studies heterogeneity was performed using the chi-square-based Cochrane Q-test, in which P-value less than 0.10 was considered significant. In addition, we have used I2 to statistically measure the heterogeneity and indicate the percentage of variance of the heterogeneity. A fixed-effect model (Mantel-Haenszel method) was used to pool ORs and 95% CI when there was no significant heterogeneity. Otherwise, a random effects model (the DerSimonian and Laird method) was used. The Pearson’s *χ*^2^ test was applied to test the Hardy-Weinberg equilibrium (HWE) in healthy controls with the significance set at P<0.05. Sensitivity analysis was performed by iteratively omitting one study at a time to determine the effects of individual study on overall data and stability of the results. Moreover, sensitivity analysis was performed by removing those studies did not in agreement with HWE in control groups. Stratification analysis was performed based on ethnicity (Caucasians, Asians, African and mixed populations), source of controls (HB or PB), genotyping methods and HWE status. The publication bias of the individual studies on *XRCC1*, *XRCC2* and *XRCC3* polymorphisms and TC risk was assessed visually inspecting the Begg’s funnel plot for asymmetry and the Egger’ linear regression test statistically. Egger`s linear regression test was used to evaluate the symmetry of the funnel plot in order to minimize the subjective influence of the visual inspection assessment, in which bias was considered with P<0.05 in Egger’s test. Statistical analyses were performed using Comprehensive Meta-Analysis (CMA) software version 2.0 (Biostat, USA). Two-sided P-values < 0.05 were considered statistically significant.

## Results

*Characteristics of included studies*

**Figure 1:**
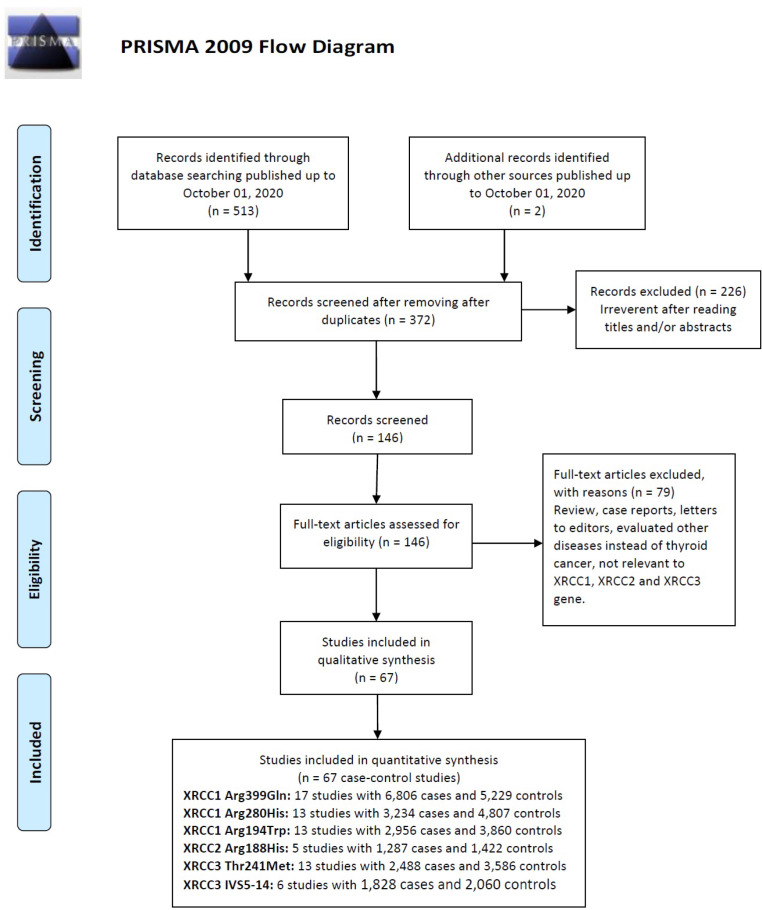
A Flow Chart Showing the Study Selection Procedure

The selection process of eligible studies is presented in Figure 1. A total of 515 potentially relevant articles were preliminarily identified though a systematic publication search. After excluding duplicate literatures and further carefully reading titles and abstracts of the remaining studies, 146 articles were performed full-text review for eligibility, among which 79 articles were excluded because were not related and did not have sufficient data. Finally, 67 case-control studies with 18,709 TC cases and 20,877 controls on the *XRCC1* (n=43), *XRCC2* (n=5) and *XRCC3* (n=19) polymorphisms met our inclusion criteria (Zhu et al., 2004, 2018; Sturgis et al., 2005; HX et al., 2006; Siraj et al., 2008; Chiang et al., 2008; Bastos et al., 2009; Ho et al., 2009; Akulevich et al., 2009; Fard-Esfahani et al., 2011; García-Quispes et al., 2011; Ryu et al., 2011; Santos et al., 2012; Fayaz et al., 2013; Wang et al., 2015; Halkova et al., 2016; Yuan et al., 2016; Yan et al., 2016; Sarwar et al., 2017; Adampourezare et al., 2017; Bashir et al., 2018). Detailed characteristics and genotype distribution of eligible studies are summarized in Table 1. Of these studies, 17 studies with 6806 cases and 5229 controls on *XRCC1* Arg399Gln, 13 studies with 3234 cases and 4807 controls on *XRCC1* Arg280His, 13 studies with 2956 cases and 3860 controls on *XRCC1* Arg194Trp, five studies with 1,287 cases and 1,422 controls on *XRCC2* Arg188His, 13 studies with 2,488 cases and 3,586 controls on *XRCC3* Thr241Met, and six studies with 1,828 cases and 2,060 controls were on *XRCC3* IVS5-14 polymorphism. Subjects in 26 of the included case-control studies were belonged to Caucasians while those in the remaining studies were Asians. Five different genotyping methods were used in these studies including PCR-RFLP, TaqMan, iPLEX Assay, MassARRAY, and ARMS-PCR. The genotype distributions in the healthy controls of 21 studies were not consistent with HWE (Table 1).

*Quantitative Data Synthesis*

*XRCC1 Polymorphisms*

**Figure 2:**
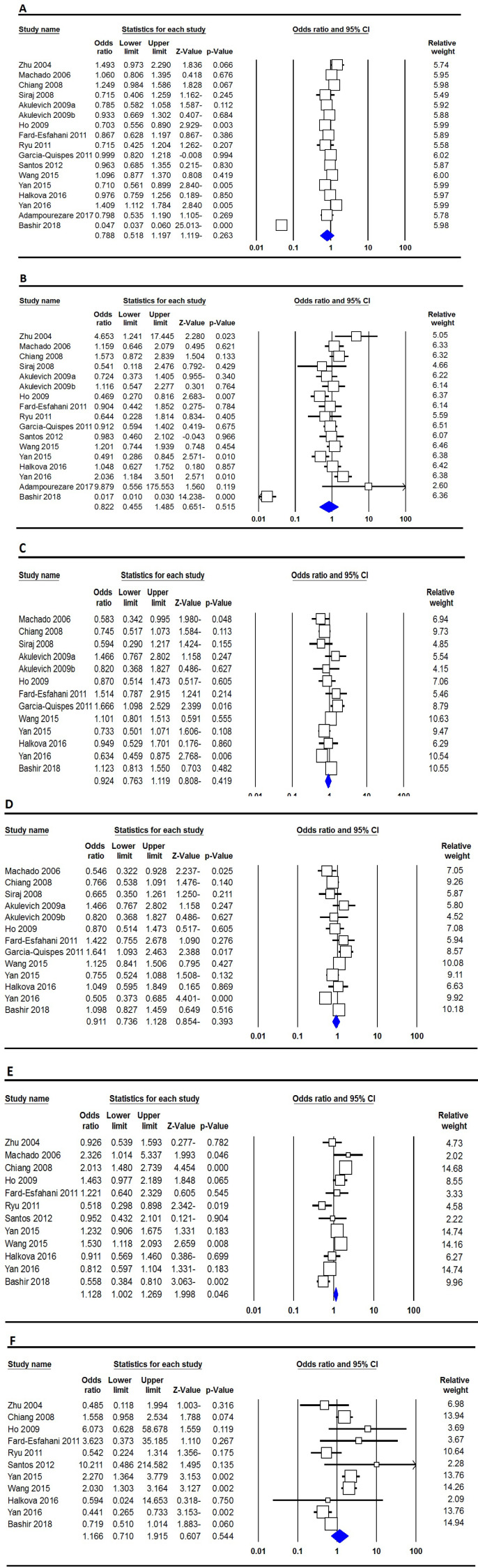
Forest Plot for the association between XRCC1 Arg188His, Arg188His and Arg194Trp Polymorphisms and TC Risk. A: Arg188His (allele model: A vs. G) B: Arg188His (homozygote model: AA vs. GG); C: Arg280His (heterozygote model: AG vs. GG); D: Arg280His (dominant model: AA+AG vs. GG); E: Arg194Trp (dominant model: TT+TC vs. CC); and F: Arg194Trp (recessive model: TT vs. TC+CC)

Table 3 presents the main results of the meta-analysis of the *XRCC1* Arg399Gln, Arg280His and Arg194Trp polymorphisms and TC risk. Pooled data revealed that the *XRCC1* Arg399Gln, Arg280His and Arg194Trp polymorphisms were not significantly associated with an increased risk of TC in the global population (Figure 2). When stratified by ethnicity, the *XRCC1* Arg399Gln polymorphism was associated with risk of TC in Caucasians under two genetic models, i.e., allele (A vs. G: OR=0.334, 95% CI 0.789-0.980, p=0.020) and dominant (AA vs. GG: OR=0.869, 95% CI 0.760-0.993, p=0.040), but not in Asians. Subgroup analyses by ethnicity still did not find a significant for association of *XRCC1* Arg280His and Arg194Trp polymorphisms and TC risk (Table 3).

*XRCC2 Polymorphism*

**Figure 3:**
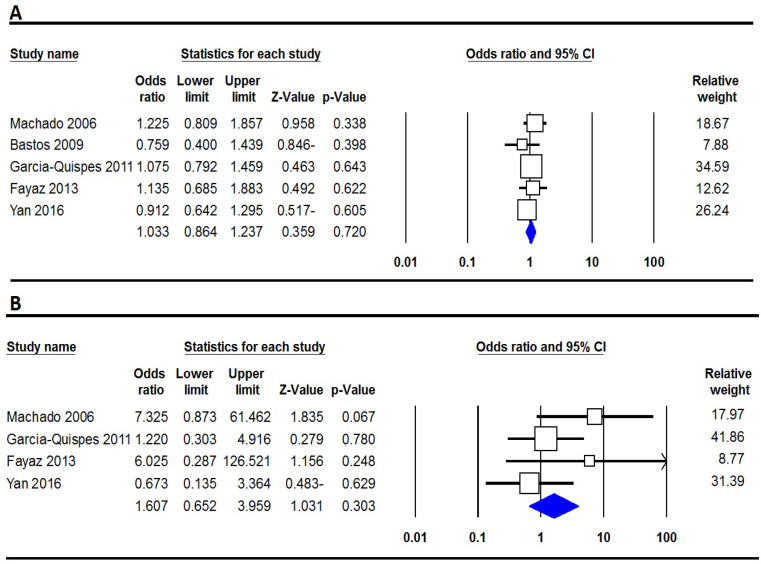
Forest Plot for the association between XRCC2 Arg188His Polymorphism and TC Risk. A, allele model (A vs. G); and B, homozygote model (AA vs. GG)

Table 2 listed the main results of the meta-analysis of *XRCC2* Arg188His polymorphism and TC risk. We pooled all the five case-control studies to evaluate the association of *XRCC2* Arg188His polymorphism with TC risk. The pooled results showed that *XRCC2* Arg188His polymorphism did not significantly associate with TC risk under all five genetic models (Figure 3). When, subgroup analyses performed according to ethnicity still did not find significant association between *XRCC2* Arg188His polymorphism and TC risk in Asians and Caucasians (Table 3).

*XRCC3 Polymorphisms*

**Figure 4.**
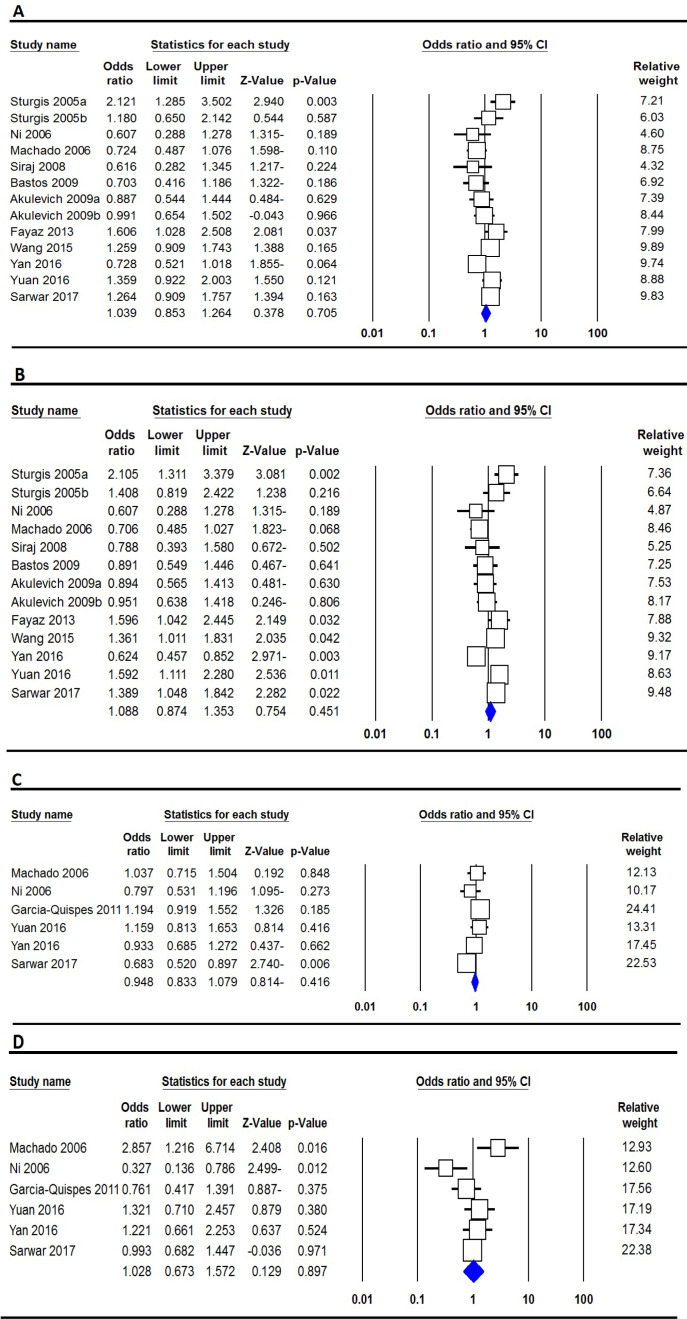
Forest Plot for the Association between *XRCC3* Thr241Met and IVS5-14 Polymorphisms and TC Risk. A, Thr241Met (heterozygote model: TC vs. CC); B, Thr241Met (dominant model: TT+TC vs. CC); C, IVS5-14 (dominant model: GG+GA vs. AA); and D, IVS5-14 (recessive model: GG vs. GA+AA).

The summary for the association of the *XRCC3* Thr241Met and IVS5-14 polymorphisms with TC risk are summarized in Table 3. Pooled data revealed that the *XRCC3* Thr241Met and IVS5-14 polymorphisms were not significantly associated with risk of TC under all five genetic models (Figure 4). When, subgroup analyses performed according to ethnicity still did not find significant association between *XRCC3* Thr241Met polymorphism and TC risk in Asians and Caucasians (Table 3).

*Test of Heterogeneity*

Significant heterogeneity existed in all of the genetic models for *XRCC1* Arg399Gln, Arg280His, Arg194Trp, *XRCC3* Thr241Met and IVS5-14 polymorphisms (Table 3). Thus, we performed subgroup analyses by ethnicity to find the possible source of heterogeneity. Results showed that Caucasians descent subjects have not overall effect on the heterogeneity, but the selected Asian descents were extremely heterogeneous.

*Sensitivity Analysis*

Sensitivity analyses were performed after sequentially removing each eligible study to assess the stability of our results. This test is regarded as an indispensable step for analyzing multiple criteria. The results showed that the significance of the pooled ORs was not influenced by any single study under all five genetic models for *XRCC1*, *XRCC2* and *XRCC3* polymorphisms, indicating that our results were highly stable. Moreover, we performed sensitivity analysis by excluding those studies did not in agreement HWE for *XRCC1* Arg399Gln, Arg280His, Arg194Trp, *XRCC3* Thr241Met and *XRCC3* IVS5-14 polymorphisms. Similarly, after excluding those studies the results indicated no significant alteration in the pooled ORs.

*Publication Bias*

**Figure 5:**
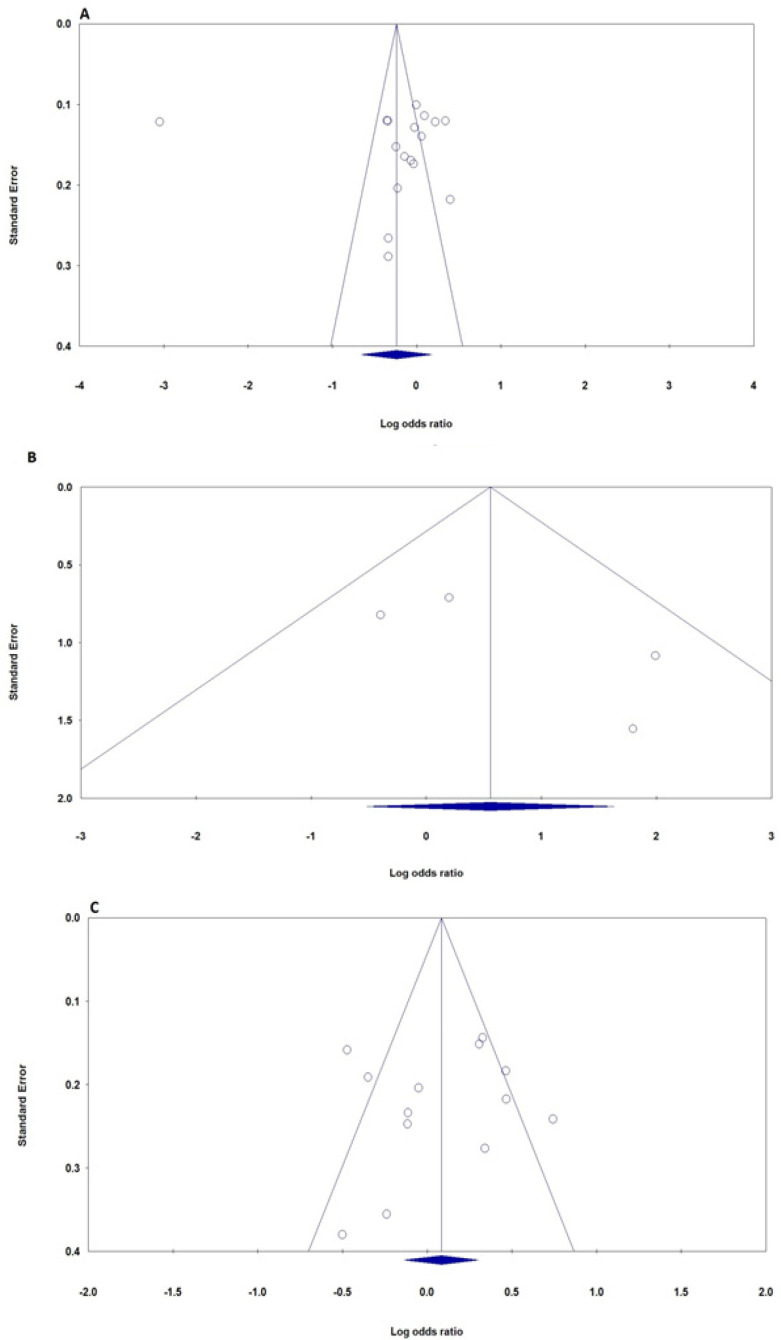
Publication Bias Test for the Association of *XRCC1, XRCC2* and *XRCC3* Polymorphisms with Risk of TC. A, *XRCC1 *Arg399Gln (allele model); B, *XRCC2* Arg188His (homozygote model); C, *XRCC3* Thr241Met (dominant model). Each point represents a separate study for the indicated association

We used the Visual inspection of funnel plot and the Egger’s weighted regression tests to assess the publication bias of eligible literatures for *XRCC1*, *XRCC2* and *XRCC3* polymorphisms and TC risk. Visual inspection of the funnel plots did not show any evidence of publication bias for *XRCC1* Arg399Gln, Arg280His, Arg194Trp, *XRCC2* Arg188His, *XRCC3* Thr241Met and IVS5-14 polymorphisms (Figure 5). Moreover, the Egger test, which was used to provide statistical evidence of funnel plot symmetry, did not show any significant publication bias in this meta-analysis (Table 3).

**Table 1.** Main Characteristics of Studies Included in the Meta-Analysis for *XRCC1 *Polymorphisms

First Author	Country (Ethnicity)	SOC	Genotyping	Case/Control	Cases					Controls					MAF	HWE
			Method		Genotype			Allele		Genotype			Allele			
Arg399Gln					GG	AG	AA	G	A	GG	AG	AA	G	A		
Zhu 2004	China(Asian)	HB	PCR-RFLP	105/105	49	44	12	142	68	57	45	3	159	51	0.243	≤0.001
Machado 2006	Spain(Caucasian)	NS	PCR-RFLP	207/251	91	88	28	270	144	113	108	30	334	168	0.335	0.592
Chiang 2008	China(Asian)	HB	TaqMan	283/469	150	110	23	410	156	277	165	27	719	219	0.233	≤0.001
Siraj 2008	KSA(Asian)	HB	PCR-RFLP	50/299	35	13	2	83	17	142	72	15	356	102	0.223	0.162
Akulevich 2009	Russia(Caucasian)	PB	PCR-RFLP	132/398	65	53	14	183	81	158	193	47	509	287	0.361	0.05
Akulevich 2009	Belarus(Caucasian)	HB	PCR-RFLP	123/199	55	50	18	160	86	75	100	22	250	144	0.365	≤0.001
Ho 2009	USA(Caucasian)	HB	PCR-RFLP	251/503	133	99	19	365	137	220	216	67	656	350	0.348	≤0.001
Fard-Esfahani 2011	Iran(Asian)	HB	PCR-RFLP	155/190	78	60	17	216	94	83	87	20	253	127	0.334	≤0.001
Ryu 2011	Korea(Asian)	HB	PCR-RFLP	111/100	87	17	7	191	31	72	19	9	163	37	0.185	≤0.001
Garcia-Quispes 2011	Spain(Caucasian)	HB	PCR-RFLP	402/479	153	186	47	492	280	196	212	66	604	344	0.363	0.476
Santos 2012	Portugal(Caucasian)	HB	PCR-RFLP	109/217	46	50	13	142	76	87	105	25	279	155	0.357	0.428
Wang 2015	China(Asian)	HB	PCR-RFLP	276/552	138	105	32	381	169	290	206	56	786	318	0.288	0.034
Yan 2015	China(Asian)	HB	iPLEX Assay	276/403	146	108	22	400	152	176	173	54	525	281	0.349	0.271
Halkova 2016	Czech(Caucasian)	HB	PCR-RFLP	209/374	97	81	31	275	143	164	160	50	488	260	0.348	0.272
Yan 2016	China(Asian)	HB	MassARRAY	403/276	176	173	54	525	281	146	108	22	400	152	0.275	0.746
Adampourezare 2017	Iran(Asian)	HB	PCR-RFLP	114/91	45	55	14	145	83	15	76	0	106	76	0.418	≤0.001
Bashir 2018	Pakistan(Asian)	HB	ARMS-PCR	3617/400	3512	89	16	7113	121	257	75	68	589	211	0.264	≤0.001
Arg280His					GG	AG	AA	G	A	GG	AG	AA	G	A		
Machado 2006	Spain(Caucasian)	NS	PCR-RFLP	207/248	183	24	0	390	24	200	45	3	445	51	0.103	0.794
Chiang 2008	China(Asian)	HB	TaqMan	283/469	224	54	5	502	64	349	113	7	811	127	0.135	0.528
Siraj 2008	KSA (Asian)	HB	PCR-RFLP	50/299	33	12	5	78	22	129	79	21	337	121	0.264	0.088
Akulevich 2009	Russia(Caucasian)	PB	PCR-RFLP	132/398	117	15	0	249	15	366	32	0	764	32	0.04	0.403
Akulevich 2009	Belarus(Caucasian)	HB	PCR-RFLP	123/195	113	10	0	236	10	176	19	0	371	19	0.049	0.474
Ho 2009	USA(Caucasian)	HB	PCR-RFLP	251/503	229	22	0	480	22	453	50	0	956	50	0.05	0.24
Fard-Esfahani 2011	Iran(Asian)	HB	PCR-RFLP	170/193	146	23	1	315	25	173	18	2	364	22	0.057	0.065
Garcia-Quispes 2011	Spain(Caucasian)	HB	PCR-RFLP	402/479	337	58	3	732	64	426	44	3	896	50	0.053	0.123
Wang 2015	China(Asian)	HB	PCR-RFLP	276/552	153	91	32	397	155	322	174	56	818	286	0.259	≤0.001
Yan 2015	China(Asian)	HB	iPLEX Assay	276/403	218	52	6	488	64	298	97	8	693	113	0.14	0.974
Halkova 2016	Czech(Caucasian)	HB	PCR-RFLP	209/374	188	19	2	395	23	338	36	0	712	36	0.048	0.328
Yan 2016	China(Asian)	HB	MassARRAY	403/370	298	97	8	693	113	218	112	40	548	192	0.259	≤0.001
Bashir 2018	Pakistan(Asian)	HB	ARMS-PCR	456/400	150	166	140	466	446	140	138	122	418	382	0.478	≤0.001
First Author	Country (Ethnicity)	SOC	Genotyping	Case/Control	Cases					Controls					MAF	HWE
			Method		Genotype			Allele		Genotype			Allele			
Arg194Trp					CC	CT	TT	C	T	CC	CT	TT	C	T		
Zhu 2004	China(Asian)	HB	PCR-RFLP	105/105	50	52	3	152	58	48	51	6	147	63	0.3	0.108
Machado 2006	Spain(Caucasian)	NS	PCR-RFLP	207/253	190	17	0	397	17	234	9	0	477	9	0.019	0.768
Chiang 2008	China(Asian)	HB	TaqMan	283/469	127	119	37	373	193	254	119	36	627	191	0.233	0.001
Ho 2009	USA(Caucasian)	HB	PCR-RFLP	251/503	203	45	3	451	51	433	69	1	935	71	0.071	0.306
Fard-Esfahani 2011	Iran(Asian)	HB	PCR-RFLP	157/187	136	18	3	290	24	166	20	1	352	22	0.059	0.641
Ryu 2011	Korea(Asian)	HB	PCR-RFLP	111/100	59	43	9	161	61	37	49	14	123	77	0.385	0.728
Santos 2012	Portugal(Caucasian)	HB	PCR-RFLP	109/217	98	8	2	204	12	196	21	0	413	21	0.048	0.453
Yan 2015	China(Asian)	HB	iPLEX Assay	276/403	124	112	40	360	192	202	173	28	577	229	0.284	0.267
Wang 2015	China(Asian)	HB	PCR-RFLP	276/552	181	52	43	414	138	411	95	46	917	187	0.169	≤0.001
Halkova 2016	Czech(Caucasian)	HB	PCR-RFLP	209/374	178	31	0	387	31	314	59	1	687	61	0.082	0.304
Yan 2016	China(Asian)	HB	MassARRAY	403/276	202	173	28	577	229	124	112	40	360	192	0.348	0.042
Adampourezare 2017	Iran(Asian)	HB	PCR-RFLP	114/91	114	0	0	228	0	91	0	0	182	0	NA	NA
Bashir 2018	Pakistan(Asian)	HB	ARMS-PCR	456/400	93	288	75	474	438	50	264	86	364	436	0.545	≤0.001

**Table 2:** Main Characteristics of Studies Included in the Meta-Analysis for *XRCC2* and *XRCC3* Polymorphisms

First Author	Country	SOC	Genotyping	Case/Control	Cases					Controls					MAF	HWE
	(Ethnicity)		Method		Genotype			Allele		Genotype			Allele			
XRCC2 Arg188His					GG	AG	AA	G	A	GG	AG	AA	G	A		
Machado 2006	Spain(Caucasian)	NS	PCR-RFLP	207/248	163	38	6	364	50	199	48	1	446	50	0.1	0.286
Bastos 2009	Portugal(Caucasian)	HB	PCR-RFLP	109/217	95	14	0	204	14	181	36	0	398	36	0.082	0.182
Garcia-Quispes 2011	Spain(Caucasian)	HB	PCR-RFLP	402/477	314	79	4	707	87	383	90	4	856	98	0.102	0.607
Fayaz 2013	Iran(Asian)	PB	PCR-HRM	171/204	141	28	2	310	32	170	34	0	374	34	0.083	0.194
Yan 2016	China(Asian)	HB	MassARRAY	403/276	324	76	3	724	82	218	55	3	491	61	0.11	0.82
XRCC3 Thr241Met					CC	CT	TT	C	T	CC	CT	TT	C	T		
Sturgis 2005	USA(Caucasian)	HB	PCR-RFLP	134/161	45	69	20	159	109	83	60	18	226	96	0.298	0.164
Sturgis 2005	USA(Caucasian)	HB	PCR-RFLP	79/161	34	29	16	97	61	83	60	18	226	96	0.298	0.164
Ni 2006	China(Asian)	NS	PCR-RFLP	191/201	179	12	0	370	12	181	20	0	382	20	0.049	0.457
Machado 2006	Spain(Caucasian)	HB	PCR-RFLP	207/248	96	88	23	280	134	94	119	35	307	189	0.381	0.786
Siraj 2008	KSA(Asian)	HB	PCR-RFLP	37/227	18	12	7	48	26	97	105	25	299	155	0.341	0.666
Bastos 2009	Portugal(Caucasian)	HB	PCR-RFLP	109/214	39	44	26	122	96	71	114	29	256	244	0.401	0.113
Akulevich 2009	Japan(Asian)	PB	PCR-RFLP	120/198	53	51	16	157	83	82	89	27	253	143	0.361	0.716
Akulevich 2009	Japan(Asian)	PB	PCR-RFLP	132/398	55	65	12	175	89	161	192	45	514	282	0.354	0.277
Fayaz 2013	Iran(Asian)	PB	PCR-RFLP	161/183	71	76	14	218	104	102	68	13	272	94	0.256	0.719
Wang 2015	China(Asian)	HB	PCR-RFLP	276/552	161	84	31	406	146	362	150	40	874	230	0.208	≤0.001
Yan 2016	China(Asian)	HB	MassARRAY	403/276	255	126	22	636	170	143	97	36	383	169	0.306	0.004
Yuan 2016	China(Asian)	HB	MassARRAY	183/367	95	64	24	254	112	232	115	20	579	155	0.211	0.254
Sarwar 2017	Pakistan(Asian)	HB	ARMS-PCR	456/400	277	109	70	663	249	273	85	42	631	169	0.211	≤0.001
XRCC3 IVS5-14					AA	AG	GG	A	G	AA	AG	GG	A	G		
Machado 2006	Spain(Caucasian)	NS	PCR-RFLP	207/248	115	74	18	304	110	140	100	8	380	116	0.233	0.048
Ni 2006	China(Asian)	NS	PCR-RFLP	181/201	83	91	7	257	105	81	98	22	260	142	0.353	0.341
Garcia-Quispes 2011	Spain(Caucasian)	HB	PCR-RFLP	398/578	236	145	17	617	179	367	179	32	913	243	0.21	0.105
Yuan 2016	China(Asian)	HB	MassARRAY	183/367	90	75	18	235	111	194	145	28	533	201	0.273	0.899
Yan 2016	China(Asian)	HB	MassARRAY	403/266	213	159	31	585	221	136	113	17	385	147	0.276	0.31
Sarwar 2017	Pakistan(Asian)	HB	ARMS-PCR	456/400	284	104	68	672	240	212	128	60	552	248	0.31	≤0.001

Abbreviations: SOC, source of control; HB, hospital based; PB, population based; NS, Not Stated; PCR, Polymerase chain reaction; RFLP, polymerase chain reaction-restriction fragment length polymorphism; ARMS, Amplification Refractory Mutation System; MAF, minor allele frequency; HWE, Hardy-Weinberg equilibrium.

**Table 3:** Summary of Meta-Analysis for the Association of *XRCC1*, *XRCC2 *and *XRCC3 *Polymorphisms with TC Risk

Subgroup	Genetic Model		Type of Model		Heterogeneity				Odds Ratio								Publication Bias			
					I^2^ (%)		P_H_		OR		95% CI		Z_test_		P_OR_		P_Beggs_		P_Eggers_	
*XRCC1* Arg399Gln																				
Overall	A vs. G		Random		97.34		≤0.001		0.788		0.518-1.197		-1.119		0.263		0.364		0.796	
	AA vs. GG		Random		92.72		≤0.001		0.822		0.455-1.485		-0.651		0.515		0.869		0.618	
	AG vs. GG		Random		92.46		≤0.001		0.725		0.511-1.030		-1.796		0.072		0.667		0.667	
	AA+AG vs. GG		Random		95.94		≤0.001		0.723		0.463-1.127		-1.433		0.152		0.216		0.767	
	AA vs. AG+GG		Random		92.05		≤0.001		0.886		0.515-1.526		-0.436		0.663		1.000		0.559	
By Ethnicity																				
Caucasians	A vs. G		Fixed		12.49		0.334		0.88		0.789-0.980		-2.327		0.02		0.548		0.442	
	AA vs. GG		Fixed		15.86		0.309		0.873		0.703-1.083		-1.234		0.217		1.000		0.892	
	AG vs. GG		Fixed		11.91		0.339		0.873		0.758-1.006		-1.871		0.061		0.548		0.234	
	AA+AG vs. GG		Fixed		18.63		0.288		0.869		0.760-0.993		-2.056		0.04		1.000		0.542	
	AA vs. AG+GG		Fixed		13.18		0.329		0.931		0.759-1.142		-0.687		0.492		0.548		0.556	
Asians	A vs. G		Random		98.42		≤0.001		0.712		0.338-1.501		-0.892		0.373		0.128		0.763	
	AA vs. GG		Random		95.66		≤0.001		0.827		0.274-2.493		-0.337		0.736		0.654		0.638	
	AG vs. GG		Random		95.46		≤0.001		0.647		0.352-1.192		-1.397		0.162		0.128		0.846	
	AA+AG vs. GG		Random		97.56		≤0.001		0.641		0.297-1.385		-1.13		0.258		0.244		0.755	
	AA vs. AG+GG		Random		95.23		≤0.001		0.903		0.324-2.517		-0.195		0.846		0.788		0.599	
*XRCC1* Arg280His																				
Overall	A vs. G		Random		75.35		≤0.001		0.914		0.740-1.128		-0.839		0.401		0.669		0.892	
	AA vs. GG		Random		67.1		0.001		0.804		0.468-1.380		-0.793		0.428		0.858		0.657	
	AG vs. GG		Random		55.29		0.008		0.924		0.763-1.119		-0.808		0.419		0.76		0.873	
	AA+AG vs. GG		Random		67.47		≤0.001		0.911		0.736-1.128		-0.854		0.393		1.000		0.779	
	AA vs. AG+GG		Random		62.74		0.004		0.828		0.506-1.355		-0.75		0.453		0.474		0.723	
By Ethnicity																				
Caucasians	A vs. G		Random		61.27		0.024		1.016		0.714-1.446		0.089		0.929		1.000		0.493	
	AA vs. GG		Fixed		42.81		0.174		1.213		0.337-4.369		0.295		0.768		1.000		0.991	
	AG vs. GG		Random		55.99		0.045		1.013		0.714-1.435		0.07		0.944		1.000		0.423	
	AA+AG vs. GG		Random		59.52		0.03		1.014		0.708-1.453		0.075		0.94		1.000		0.45	
	AA vs. AG+GG		Fixed		40.56		0.186		1.185		0.329-4.268		0.26		0.795		1.000		0.982	
Asians	A vs. G		Random		81.92		≤0.001		0.853		0.651-1.118		-1.149		0.251		1.000		0.817	
	AA vs. GG		Random		74.64		0.001		0.757		0.422-1.357		-0.935		0.35		0.367		0.485	
	AG vs. GG		Random		55.62		0.035		0.868		0.691-1.090		-1.22		0.222		0.548		0.986	
	AA+AG vs. GG		Random		72.46		0.001		0.848		0.648-1.109		-1.204		0.229		0.367		0.975	
	AA vs. AG+GG		Random		70.92		0.002		0.791		0.467-1.340		-0.873		0.382		0.367		0.558	
*XRCC1 *Arg194Trp																				
Overall	T vs. C		Random		83.16		≤0.001		1.121		0.888-1.416		0.959		0.337		0.583		0.693	
	TT vs. CC		Random		82.74		≤0.001		1.155		0.631-2.116		0.468		0.64		0.937		0.815	
	TC vs. CC		Random		68.86		≤0.001		1.057		0.834-1.340		0.458		0.648		1.000		0.693	
	TT+TC vs. CC		Fixed		77.69		≤0.001		1.087		0.836-1.415		0.623		0.533		1.000		0.621	
	TT vs. TC+CC		Random		77.52		≤0.001		1.166		0.710-1.915		0.607		0.544		0.937		0.611	
By Ethnicity																				
Caucasians	T vs. C		Fixed		38.71		0.18		1.28		0.992-1.652		1.896		0.058		0.734		0.73	
	TT vs. CC		Fixed		0.00		0.389		4.031		0.828-19.62		1.726		0.084		1.000		0.649	
	TC vs. CC		Fixed		42.14		0.159		1.204		0.915-1.585		1.326		0.185		1.000		0.928	
	TT+TC vs. CC		Fixed		38.96		0.178		1.251		0.955-1.639		1.623		0.105		0.734		0.822	
	TT vs. TC+CC		Fixed		0.00		0.396		3.966		0.815-19.29		1.707		0.088		1.000		0.668	
Asians	T vs. C		Random		88.07		≤0.001		1.058		0.794-1.409		0.385		0.700		0.457		0.977	
	TT vs. CC		Random		86.9		≤0.001		1		0.528-1.894		0.000		1.000		1.000		0.79	
Subgroup		Genetic Model		Type of Model		Heterogeneity				Odds Ratio								Publication Bias		
						I^2^ (%)		P_H_		OR		95% CI		Z_test_		P_OR_		P_Beggs_		P_Eggers_
By Ethnicity																				
Asians		TC vs. CC		Random		76.19		≤0.001		1		0.740-1.350		-0.002		0.999		0.804		0.421
		TT+TC vs. CC		Random		83.98		≤0.001		1.017		0.724-1.429		0.098		0.922		0.62		0.344
		TT vs. TC+CC		Random		82.51		≤0.001		1.051		0.629-1.754		0.188		0.851		1.000		0.981
*XRCC2* Arg188His																				
Overall		A vs. G		Fixed		0.00		0.694		1.033		0.864-1.237		0.359		0.72		0.806		0.671
		AA vs. GG		Fixed		24.08		0.267		1.607		0.652-3.959		1.031		0.303		0.734		0.245
		AG vs. GG		Fixed		0.00		0.918		0.968		0.794-1.180		-0.322		0.748		0.462		0.136
		AA+AG vs. GG		Fixed		0.00		0.856		0.998		0.822-1.212		-0.017		0.986		0.22		0.398
		AA vs. AG+GG		Fixed		24.34		0.265		1.601		0.650-3.941		1.024		0.306		0.734		0.238
*XRCC3 *Thr241Met																				
Overall		T vs. C		Random		91.8		≤0.001		1.119		0.823-1.521		0.715		0.475		0.951		0.579
		TT vs. CC		Random		69.21		≤0.001		1.217		0.869-1.705		1.144		0.253		0.837		0.933
		TC vs. CC		Random		59.92		0.003		1.039		0.853-1.264		0.378		0.705		0.854		0.489
		TT+TC vs. CC		Random		72.16		≤0.001		1.088		0.874-1.353		0.754		0.451		0.502		0.519
		TT vs. TC+CC		Random		69.72		≤0.001		1.264		0.919-1.736		1.442		0.149		0.837		0.897
By ethnicity																				
Asians		T vs. C		Random		93.27		≤0.001		1.149		0.768-1.719		0.675		0.499		0.348		0.527
		TT vs. CC		Random		73.5		≤0.001		1.127		0.730-1.740		0.541		0.588		0.386		0.707
		TC vs. CC		Random		51.54		0.036		1.047		0.851-1.287		0.432		0.666		0.465		0.256
		TT+TC vs. CC		Random		72.36		≤0.001		1.067		0.828-1.374		0.498		0.618		0.117		0.275
		TT vs. TC+CC		Random		78.73		0.003		1.153		0.697-1.906		0.554		0.58		0.710		0.88
Caucasians		T vs. C		Random		85.57		0		1.04		0.673-1.609		0.178		0.859		0.734		0.33
		TT vs. CC		Random		66.18		0.031		1.426		0.789-2.578		1.175		0.245		0.089		0.102
		TC vs. CC		Random		77.33		0.004		1.052		0.624-1.775		0.191		0.848		0.734		0.581
		TT+TC vs. CC		Random		77.77		0.004		1.19		0.729-1.942		0.694		0.488		0.734		0.271
		TT vs. TC+CC		Fixed		56.22		0.077		1.367		0.997-1.874		1.94		0.052		0.734		0.484
*XRCC3* IVS5-14																				
Overall		G vs. A		Fixed		52.71		0.061		0.97		0.875-1.075		-0.586		0.558		0.259		0.269
		GG vs. AA		Random		63.84		0.017		0.996		0.646-1.537		-0.018		0.986		0.452		0.798
		GA vs. AA		Random		61.03		0.025		0.927		0.741-1.160		-0.663		0.507		1.000		0.708
		GG+GA vs. AA		Fixed		53.23		0.058		0.948		0.833-1.079		-0.814		0.416		0.707		0.292
		GG vs. GA+AA		Random		64.21		0.016		1.028		0.673-1.573		0.129		0.897		0.707		0.985

## Discussion

Human *XRCC1* gene is mapped to chromosome 19q13, composed of 17 exons and spans approximately 31.9kb (Li et al., 2013). The *XRCC1* protein has no known catalytic activity, but serves an important component of the base excision repair (BER) pathway via its role as a central scaffolding protein physically associated with DNA ligase III at its COOH terminus (Li et al., 2012). More than 300 validated polymorphisms in the human *XRCC1* gene are listed in the dbSNP database, of which, the most extensively studied SNPs are Arg399Gln (exon 10), Arg280His (exon 9) and Arg194Trp (exon 6) polymorphisms in different cancer (Li et al., 2012, Li et al., 2013; Qi et al., 2014). Our results revealed that the *XRCC1* Arg399Gln, Arg280His and Arg194Trp polymorphisms were not significantly associated with risk of TC in the global population. However, subgroup analysis showed that the *XRCC1* Arg399Gln polymorphism was associated with risk of TC in Caucasians, but not in Asians. To date, several meta-analyses have been performed to undertake the association of polymorphisms in *XRCC1* in development of TC.

Human *XRCC2* gene is paralogue of RAD51 plays a pivotal role in the homologous recombination repair (HRR) machinery, maintenance of the genome integrity and the control of genomic rearrangement processes causes to the chromatid breaks (Kamali et al., 2017). *XRCC2* gene is located on human chromosome 7q36.1, consists of three exons, which are distributed 29 DNA repair over a 30 kb region. In exon 3, an Arg188His polymorphism (rs3218536) has been identified on the coding region of *XRCC2* as potential cancer susceptibility loci in recent studies, although association results are controversial. However, the potential phenotypic effects of this polymorphism are currently unknown. Previous epidemiological studies that examined the *XRCC2* Arg188His polymorphisms with TC risk have provided controversial results. For example, Yan et al., (2020) reported that there was no significant association between *XRCC2* Arg188His polymorphism and TC risk in a Chinese population. However, Fayaz et al., reported that *XRCC2* Arg188His polymorphism is associated with an increased risk of TC in an Iranian population. To the best of our knowledge, this was the first meta-analysis to evaluate association of the *XRCC2* Arg188His polymorphism with TC risk. Our results revealed that there was no significant association between *XRCC2* Arg188His polymorphism and TC risk in the overall population.

Human *XRCC3*, also known as CMM6, is a member of the RecA/Rad51-related protein family that participates in HRR to maintain chromosome stability which was originally identified by its ability to complement the DNA repair defect (Duarte et al., 2005; Sobhan et al., 2017). Human *XRCC3* gene is located on chromosome 14q32.3, contains 10 exons (its seven exons lie in the region taking 13.5 kbp) and spans 21 kbp length (Ali et al, 2016; Liu et al, 2019). In this meta-analysis, our pooled data showed that the *XRCC3* IVS5-14 and Thr241Met polymorphisms were significantly associated with an increased risk of TC in the overall population. Moreover, subgroup analysis showed that there was no a significant association between the *XRCC3* IVS5-14 and Thr241Met polymorphisms and an increased risk of TC. Unlike our results, Lu et al., in a meta-analysis of eight studies with 963 TC cases and 1,942 controls reported that the *XRCC3* Thr241Met polymorphism was associated with the risk of TC in the global population, but they did not observe significant association in by ethnicity (Lu et al., 2015). On the basis of availability of five more studies with 2,589 cases and 3,596 controls on *XRCC3* Thr241Met polymorphism and TC, our results more reliable and powerful results than the previous meta-analysis.

The present meta-analysis has some novelty and advantages. First, to the best of our knowledge, this study was the first meta-analysis to comprehensively evaluate the association of *XRCC2* Arg188His polymorphism with susceptibility to TC. Second, our results were inconsistent with the previous meta-analysis on *XRCC3* Thr241Met polymorphism association with TC risk might be due to including large sample size. Finally, no publication bias was found in the present study and sensitivity analysis also indicated that no single study yield obvious impact on the pooled results, which indicating that the results of the present meta-analysis are statically robust.

Despite above mentioned advantages, the current meta-analysis has some limitations which should be addressed. First, the sample size is still relatively small, which might not enough statistical power to explore the real association of the *XRCC2* Arg188His and *XRCC3* IVS5-14 polymorphisms with TC risk, which leads to the improper publication bias for these polymorphisms. Second, in the meta-analysis all of the included studies were on the Caucasian and Asians, and there was no study in African and mixed populations among the eligible studies. Therefore, need to further studies on a large scale on African and mixed populations to verify this result. Third, the study might have experienced the publication bias due to the inclusion of English and Chinese literature, which could have limited the published evidences. Fourth, the control group of several studies was not in accordance with HWE, which may be attributed to the reason as genotyping error. However, deletion of those studies did not change the results of quantitative synthesis, suggesting the robustness of results. Fifth, our pooled ORs were based on un-adjusted data for potential covariates such as age, sex, lifestyle, exposure and environmental factor, which might have affected the accuracy of the results, though no sufficient information available for most of studies included in the meta-analysis. Finally, TC is a multi-factorial disease from complex interactions between environmental factors and genetic factors. In this meta-analysis, we had insufficient data to conduct an evaluation of such interactions for the role of *XRCC1*, *XRCC2* and *XRCC3* polymorphisms and factors in TC development.

In summary, the present meta-analysis suggested that the *XRCC1* Arg399Gln, Arg280His, Arg194Trp, *XRCC2* Arg188His, *XRCC3* Thr241Met and IVS5-14 were not significantly associated with an increased risk of TC in global population. However, subgroup analyses by ethnicity showed that the *XRCC1* Arg399Gln polymorphism was associated with risk of TC in Caucasians, but not in Asians. Taking into account the aforementioned limitations, further studies are highly needed in the future.

## Author Contribution Statement

Conceived and designed the study and experiments: MM, SAD, SMT. Performed the experiments: FA and JJN

Analyzed the data: HN and SAD. Contributed reagents/materials/analysis tools: SHS and SK. Wrote the paper: HN, MM and FA. All authors reviewed the manuscript.
